# Reflective color filter with precise control of the color coordinate achieved by stacking silicon nanowire arrays onto ultrathin optical coatings

**DOI:** 10.1038/s41598-019-40001-1

**Published:** 2019-03-04

**Authors:** Han Sung Song, Gil Ju Lee, Dong Eun Yoo, Yeong Jae Kim, Young Jin Yoo, Dong-Wook Lee, Vantari Siva, Il-Suk Kang, Young Min Song

**Affiliations:** 1 School of Electrical Engineering and Computer Science, Gwangju Institute of Science and Technology (GIST), 123 Cheomdangwagi-ro, Buk-gu, Gwangju, 61005 Republic of Korea; 2 National Nanofab Center, Korea Advanced Institute of Science and Technology, 291 Daehak-ro, Yuseong-gu, Daejeon 34141 Republic of Korea

## Abstract

The engineering of structural colors is currently a promising, rapidly emerging research field because structural colors of outstanding spatial resolution and durability can be generated using a sustainable production method. However, the restricted and saturated color range in micro/nano-fabricated structural ‘pigments’ has hindered the dissemination of structural color printing. Here, this article presents a spectral mixing color filter (SMCF), which is the concept of fine-tunable color systems, capable of addressing the current issues in structural color engineering, by stacking a vertical silicon nanowire array embedded in a transparent polymer onto ultrathin optical coating layers. These two photonic structures enable independent tuning the optical resonance of each structure, depending on geometrical parameters, such as the diameter of nanowires and thickness of absorbing medium. Hence, the SMCF facilitates the linear combination of two resonant spectra, thereby enabling fine-tuning and widening of the color gamut. Theoretical studies and experimental results reveal the detailed working mechanisms and extraordinary mechanical feature of the SMCF. Based on the analyses, the concept of flexible optical device, *e*.*g*., a reflective anti-counterfeiting sticker, is demonstrated. Successful characterization demonstrates that the proposed strategy can promote the color controllability/purity of structural color and the applicability as flexible optical device.

## Introduction

Structural color can be generated by the interaction of the incident light with resonances in the nanostructures of materials^1–3^. These nanostructural color generations have been widely emerged as a key alternative to the use of traditional dyes or pigments due to their conspicuous advantages in terms of environment, compactness, spectral selectivity, low photodegradation, and high stability^4^. Specifically, recycling of conventional colored materials is significantly difficult because of the problems related to the dissociation of chemical compounds in the colored objects^2^. However, structural color offers the advantages of easier recycling, higher reproduction fidelity, and reduction of the number of the necessary materials^5,6^. Moreover, compared to the traditional pigment-based color printing, structural color printing enables a remarkably higher printing spatial resolution (~two-orders of magnitude)^7^. The resonant wavelength of such nanoscale coloration can be tuned by controlling the geometrical parameters of the building blocks, such as their size^8–15^, shape^10,16^, period^12,13,17–19^, and thickness^20–24^. Among the various candidates for nanoscale coloration, silicon (Si) nanostructures are considered as the most suitable candidates because of the low-cost/mature fabrication process and high color purity^25–30^ resulted from the low absorption loss of Si. Because of these apparent advantages of Si nanostructures, they have widely been used in many color elements, for example, vertical Si nanowire arrays (Si NWAs), Si nanoantennas, and thin-film configurations^16,22,24–30^. In particular, in Si NWAs, successful demonstrations of multicolor generations and multispectral imaging have been reported^26–28^. Various numerical studies have found that the optical phenomena of sparse and dense Si NWAs originate from the leaky/guided mode and the Bloch mode, respectively^31,32^. Due to the supports of both optical resonant modes, Si NWAs exhibit the intriguing optical features, such as a strong wavelength-selectivity and unity absorption, depending on the Si nanowire diameter.

Recently, the aforementioned advantages of Si NWAs have motivated the development of nanoscale color printing techniques. However, the spread of nanoscale color printing has been inhibited by their inability to compete with the saturated and wide gamut of traditional pigments. Here, we propose a new class of color filters for widening/fine-tuning of the color gamut based on the interaction of light resulting from stacked color filters, such as polydimethylsiloxane (PDMS) embedded vertical Si NWA and an absorbent ultrathin film (*e*.*g*., a-Si thin-films coated on Ag layer). In this stacked filter, each optical resonance is achieved using the diameter-dependence of the guided modes of the Si NWA and the thickness-dependence of the destructive interference in the a-Si thin-films on Ag^21–24^. In particular, unlike color mixing and multilayer stacks based on plasmonic nanostructures, our spectral mixing color filter (SMCF) produces a linear combination of reflectance spectra^33,34^. The softness of each photonic configuration facilitates a flexible SMCF enabling novel application concepts for flexible optical device such as an optical anti-counterfeiting sticker. To examine the wavelength selectivity of SMCF according to the underlying filter, we first analyze the reflectance of Si NWAs on different substrates (*e*.*g*., Ag and perfect absorber; PA). We also perform a theoretical analysis and experimental validation on SMCF to investigate the principle of the color mixing effect and mechanical softness. Finally, the concept of the anti-counterfeiting sticker is demonstrated by fabricating SMCF on a flexible polymer film.

## Results

**Figure 1:**
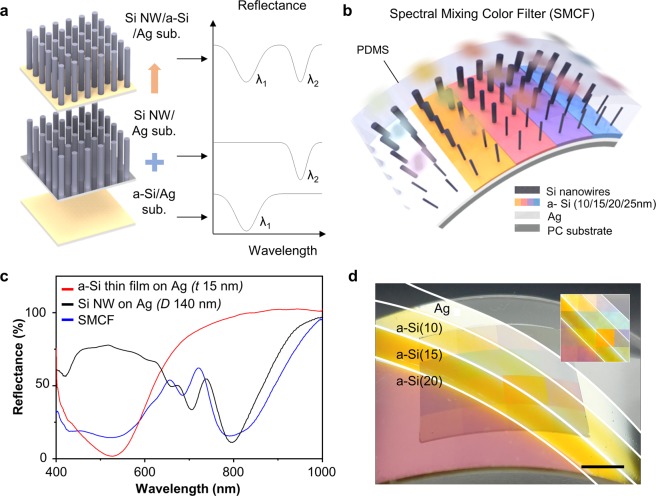
(**a**) Schematic illustration of spectral mixing color filter (SMCF) by combining two different photonic structures such as Si nanowire arrays (Si NWAs) on Ag and amorphous silicon (a-Si) on Ag. (**b**) Conceptual art of flexible SMCF by fabricating on a polycarbonate substrate (PC). (**c**) Measured reflectance spectra of the a-Si 15 nm on Ag thin film 80 nm (red line), Si NWA with diameter 140 nm (black line) and the combined structure, SMCF (blue line). (**d**) Photograph of the flexible SMCF with stress via bending and without stress (inset). Squares (5 × 5 mm^2^) indicate Si NWAs with the different diameters ranging from 80 to 170 nm with 10 nm step from the left-top to the second square from the bottom. The remaining Si NWAs have different periods and diameters. The scale bar is 5 mm.

Figure 1a,b show the schematic illustrations of the proposed color system, *i*.*e*., SMCF, composed of a vertical Si NWA and an ultrathin a-Si layer on highly reflective substrate (*e*.*g*., silver layer). Both optical filters provide different reflectance dips depending on their structural resonances, which are linearly refined by the thickness and diameter of the a-Si thin film and Si nanowire, respectively (Figs S1 and S2). The Si NWA layer selectively rejects the incident photon, whereas the a-Si layer absorbs the light broader spectral range. Such selective/broad range light-rejection can variously tune the overall reflectance spectra by stacking the Si NWA layer and the ultrathin color film. The SMCF is covered with a transparent polymer, PDMS, because the Si NWA is embedded into the PDMS to transfer the Si NWA onto a-Si thin film layer from Si substrate (Fig. 1b). The PDMS scarcely exhibits the effect on the optical resonance of the two photonic structures. Scanning electron microscopy (SEM) images display the fabricated Si NWAs with various diameters before applying the PDMS coating (Fig. S3). Although the Si NWA is dominantly obeyed by the diameter, the influences of period and height should be considered. In the period, a sweet spot exists to eliminate the incident light, whereas, in the case of height, a longer wire is effective to reduce the reflection. However, since the taller Si NWA is more difficult to fabricate, we determined the height and period of Si NWA through the parametric optimization (Fig. S4). All nanowires were of 2 μm in height and were distributed in an array with the period of 1.25 μm in each square. The fabrication process of SMCF is described in Methods and the Supporting information (Figs S5 and S6).

The Si NWA with a diameter of 140 nm (*D*140) on the Ag substrate has a dip in the reflectance spectra at 770 nm wavelength corresponding to the HE_11_ mode of Si NWA (Fig. 1c; black). The dip in the reflectance of the PDMS coated 15-nm a-Si on Ag (*t*15) is observed at the wavelength of 520 nm (Fig. 1c; red). In SMCF, the linear combination of two reflectance spectra is observed (Fig. 1c; blue). Figure 1d displays the photograph of the proposed color systems on the bent polycarbonate (PC) film, generating a variety of colors depending on the thickness of the a-Si layer (*i*.*e*., 0 nm, 10 nm, 15 nm, and 20 nm) and the diameter of Si nanowires (*i*.*e*., 80 to 170 nm with the 10 nm step). The diagonal white solid lines separate the layers with a difference in the thickness of a-Si. The squares (5 × 5 mm2) in Fig. 1d are the stacked Si NWA with a variation of diameter from 80 to 170 nm. The softness of SMCF exhibits that SMCF maintains the pristine resonant wavelengths even in bending status (Fig. S7), hence the SMCF is a potential candidate for applications of optical devices in flexible forms.

**Figure 2:**
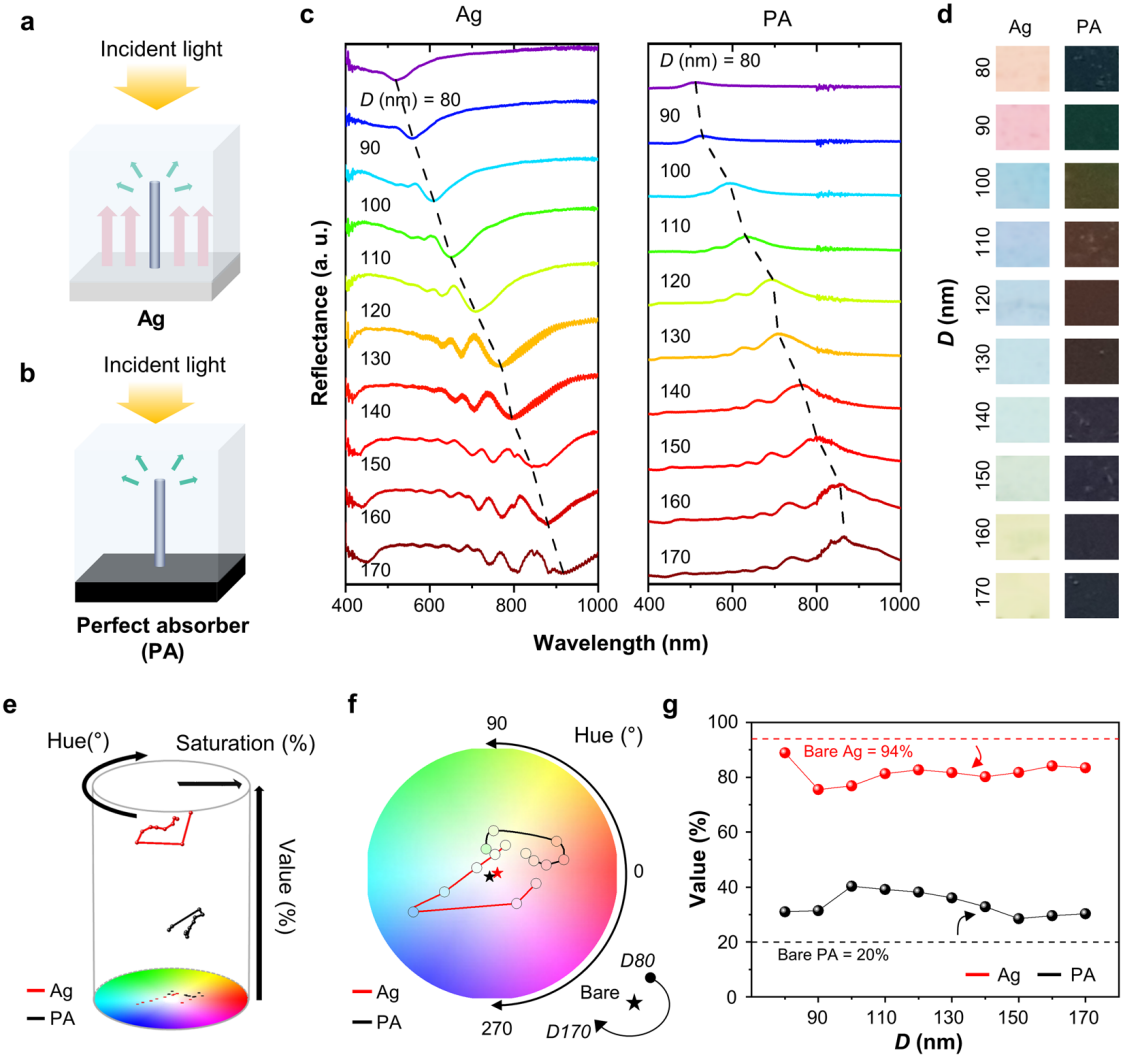
Schematic illustration of the light path in the Si NWA with a difference of the substrate, (**a**) Ag substrate and (**b**) PA substrates. (**c**) Measured reflectance spectra of the Si NWAs with a diameter ranging from 80 to 170 nm on (left) Ag and (right) PA substrates. (**d**) Photographs of the Si NWAs on Ag and PA substrates. (**e**) 3D HSV (e.g., Hue, Saturation, and Value) of Si NWAs on Ag and PA substrates. (**f**) The top view of (**e**) with a color map. (**g**) The Value information of the Si NWA as a function of the diameter on different substrates. The bare Ag and PA have the Values of 94% and 20%, respectively.

Since the SMCF is employed by transferring the Si NWA onto a-Si/Ag layer that is a reflective filter, the reflectance spectrum of underlying layer serves as a base reflectance of the overall color system. Therefore, the reflectance of bottom layer is imperative to determine the optical characteristics and color reproduction of SMCF. To analyze the effect of the substrate in SMCF, the reflectance spectra of the Si NWAs on different substrates are measured and quantitatively characterized in terms of color science, depending on the brightness of substrate (Fig. 2a,b). The Si NWAs on high reflective substrate, *i*.*e*., Ag layer, display consecutive ‘resonance dip’ along the variation of diameter (Fig. 2c; left). The photons with the wavelength corresponding to the HE_11_ mode of Si NWA are vanished, while the photons not corresponding to guided modes are reflected. The resonances by the Si NWA on Si substrate are identical to the case of Ag substrate, but the intensity of resonance is too weak (Fig. S8). On PA substrate, the reflectance spectra show ‘resonance peak’ at the wavelengths of HE_11_ mode while the light at the other wavelengths is absorbed (Fig. 2c; right). As a result, the reflectance spectra of the Si NWA on Ag and PA substrates show contradictory properties to each other. Since the wavelength of these guided modes follows a similar trend, the resonant wavelengths are almost same (Fig. S9). Figure 2d compares the color of the Si NWAs on Ag and PA with different diameters (the original pictures are in Fig. S9).

For quantitative comparison in terms of the color science, the measured reflectance spectra are reconstructed into a Hue, Saturation, and Value (HSV) color map (Fig. 2e). Figure 2f shows a 2D plot with the variation of Hue and Saturation, *i*.*e*., the top view of the HSV color map. With increasing diameters of the Si NWAs on both substrates, their Hues were found to decrease. The Hue values range from 348° (*D*80) to 67° (*D*170) for the Si NWA on the Ag film. For the Si NWA on the PA, the range of Hue is from 105° (*D*80) to 9° (*D*130), which is not covered by the Si NWA on Ag as the subtractive color filter. Figure 2g represents the Values (*i*.*e*., brightness) as a function of the diameter of the Si NWAs. The trend of the Value of the sample strongly depends on the reflectance of the substrates, *i*.*e*., the Value enhances when the reflectance of substrate increases. In the cases of Ag substrate, the stacked structures function a subtractive color filter, hence it lowers the Value compared to those of bare substrates (*i*.*e*., 94%), whereas the Si NWA on PA substrate enhances the Value of PA (*i*.*e*., 20%) because the overall structures possess an additive color filter feature. Therefore, these results demonstrate that the reflectivity of the underneath plays a key role in tuning the color of Si NWAs regarding Hue and Value.

**Figure 3:**
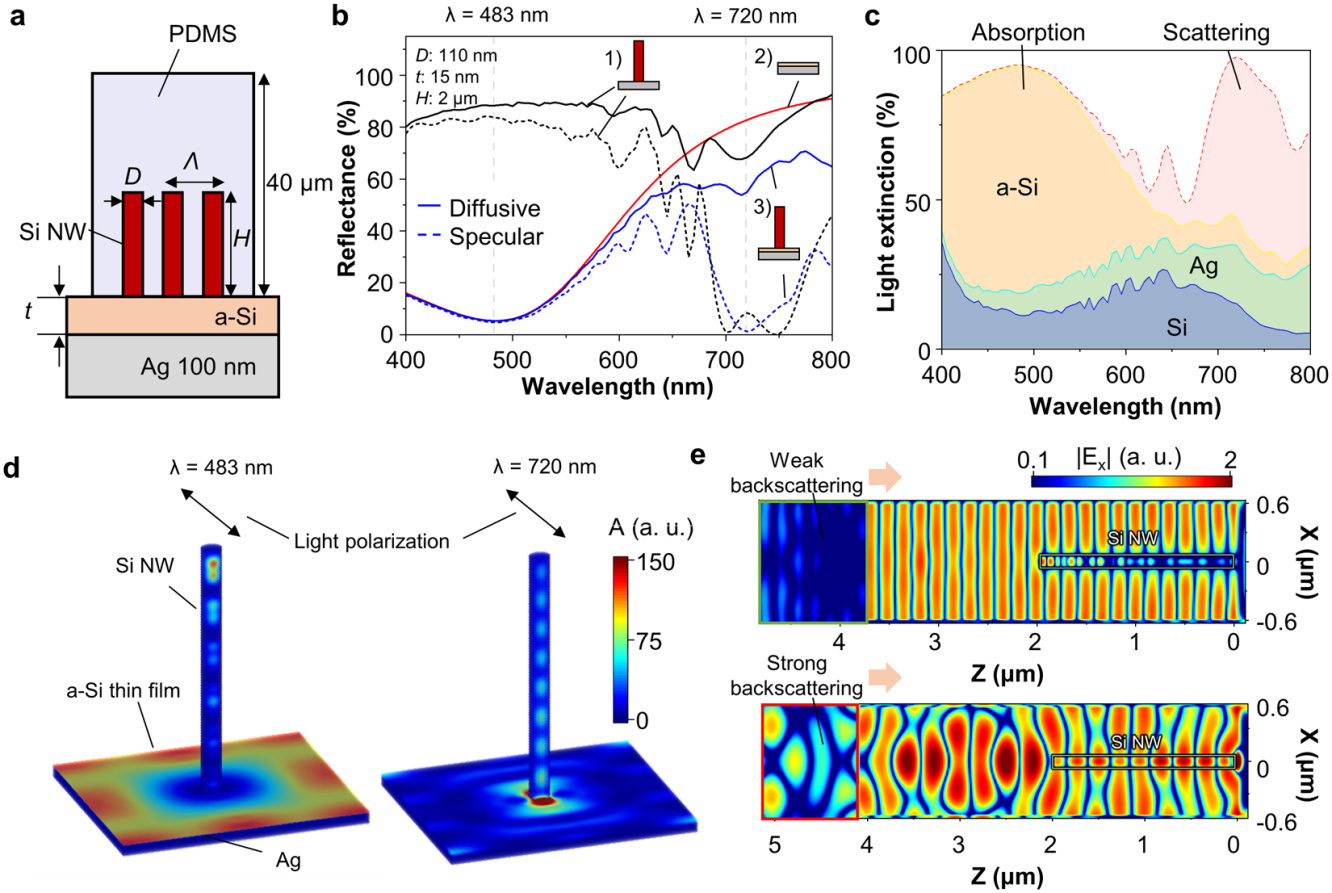
(**a**) Schematic illustration of SMCF with geometrical parameters as follows: *D* = 110 nm, *H* = 2 μm, *Λ* = 1.25 μm, and *t* = 15 nm. (**b**) Simulated reflectance spectra of three photonic structures: (1) Si NWA on Ag, (2) a-Si thin film on Ag, and (3) SMCF. Solid and dashed lines indicate that the diffusive and specular reflectance spectra, respectively. (**c**) Stacked area of light extinction ratio depending on the wavelength. The absorption, which is one type of light extinction, is distinguished with materials such as a-Si, Ag, and Si. (**d**) 3-dimensional absorption profiles and (**e**) E-field distributions of SMCF at two resonant wavelengths (i) absorption-based resonance; 483 nm and (ii) scattering-based resonance; 720 nm. The orange arrow indicates the light propagation direction. The detailed description for the scattered E-field simulation is depicted in Methods and Fig. S7.

Figure 3 computationally reveals the resonance principles of the SMCF. Figure 3a illustrates the schematic of SMCF with the geometrical parameters as follows: *D* = 110 nm, *H* = 2 μm, *Λ* = 1.25 μm, and *t* = 15 nm. The thicknesses of PDMS and Ag layer were fixed as 40 μm and 100 nm, respectively. Figure 3b shows the diffusive and specular reflectance spectra of 1) Si NWA on Ag substrate, 2) a-Si thin film on Ag substrate, and 3) SMCF, respectively. This result demonstrates that two resonances, based on multiple interferences and HE_11_ waveguide mode, differently extinct the incident light. In a-Si thin film/Ag (Fig. 3b; red line), the reflectance dip is found at the wavelength of 483 nm, and the diffusive and specular reflectance curves are identical to each other meaning the incident light is perfectly absorbed without any scattering. For Si NWA on Ag (Fig. 3b; black lines), the specular reflectance has a dip at the wavelength of 720 nm, whereas optical resonances are hardly observed in the diffusive reflectance. This difference between specular and diffusive reflectance indicates that the resonance of Si NWA/Ag is based on strong scattering. Light extinction ratio of the stacked layer (Fig. 3c) also support the fact that the SMCF simultaneously presents both absorption-/scattering-based resonances at two different wavelengths.

Figure 3d,f visualize these absorption-/scattering-based optical resonances by 3D finite-difference time-domain (FDTD) method (See also Fig. S10). At the wavelength of 483 nm, the dominant absorption occurs at the surface of the a-Si thin film except for the Si nanowire standing spot (Fig. 3d; left). This result is in line with the light extinction ratio depicted in Fig. 3c. However, at the wavelength is 720 nm, strong light absorption is observed only in the polarization direction of the field at the bottom of the Si nanowire (Fig. 3d; right). This extremely localized absorption at the bottom side of Si nanowires is not sufficient to eliminate the incident photon, but the Si nanowires act as a strong scatter shown in Fig. 3e. The electric field distributions in boxed areas show the scattered light in backward direction. This result shows that the weak backscattering is found at absorption-based resonant wavelength, whereas the light is strongly scattered in backward at scattering-based resonant wavelength. In addition to Si, other materials can be used as a scatter or absorber in vertically aligned nanowire array configuration. A material with higher refractive index shows a resonance at the longer wavelength with the same diameter (Fig. S11a). Depending on the applications, scattering-/absorption-dominant resonance could be realized by selecting lossy or lossless medium. For example, TiO_2_ or GaAs is each option to implement scattering or absorption resonant nanowire arrays (Figs S11b and S11c).

**Figure 4:**
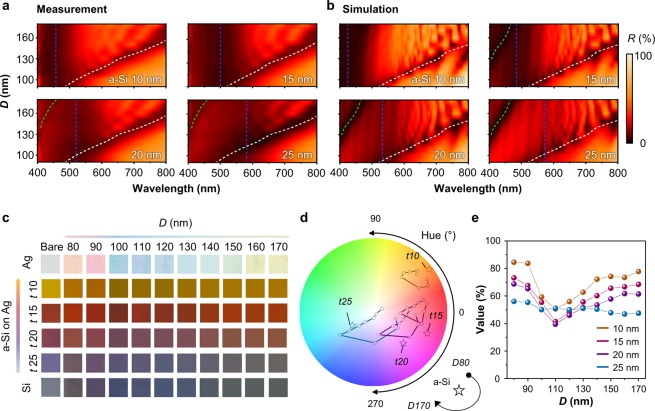
The (**a**) measured and (**b**) simulated contour plots of the SMCF as functions of the wavelength and the diameter of Si NWA with the change of thickness of a-Si from 10 to25 nm. Dashed lines indicate the resonant wavelengths. (*i*.*e*., White line: HE_11_ mode, green line: HE_12_ mode, and blue line: interference in a-Si thin film) (**c**) The color pallet with the photographs of the fabricated SMCF depending on the thickness and diameter of a-Si and Si NWA, respectively. (**d**,**e**) HSV (*i*.*e*., Hue, Saturation, and Value) of SMCF on a-Si optical coating with different thicknesses (*i*.*e*., 10, 15, 20, and 25 nm). (**d**) The top view of HSV representing Hue and Saturation values. The symbol of ‘Star’ marks the color representation of only a-Si optical coating. (**e**) The Value information of the SMCF as a function of the diameter and on thickness of Si NWA and a-Si optical coating, respectively.

Figure 4a,b represent the contour maps of measured/simulated reflectance of the SMCF as functions of diameter and wavelength for the different thicknesses of the a-Si layers. The dashed lines indicate the reflectance dips at the wavelengths corresponding to absorption-/scattering-based resonances (*i*.*e*., blue and white dashed lines). Upon increasing the thickness of the a-Si layer from 10 to 25 nm, the location of the dip was found to shift towards the longer wavelengths (from 457 to 581 nm). The dips for the scattering have the same tendency for the diameters of the Si NWA. Interestingly, another mode, namely, the HE_12_ mode (*i*.*e*., green dashed line), starts appearing for the thicknesses of a-Si layers above 20 nm (Fig. 4a)^31^. The absorption-based resonance and HE_12_ modes fall at the similar wavelength regime for the lower thicknesses (10–15 nm) of the a-Si layer. The simulation result also supports that the HE_12_ mode is veiled at extremely thin a-Si thickness (Fig. 4b). In the simulation results, at the a-Si thickness of 10 nm, the HE_12_ mode resonance is not observed, and even at 15 nm, this resonance is very weakly found. In addition to the weak resonance, the general Si photodetector has a low sensitivity around UV range (*i*.*e*., 400 nm). Thus, the strong resonance by a-Si thin film around the wavelength of 400 nm disturbs the detection of HE_12_ mode in measurement. Except for the HE_12_ mode, the measured and simulated reflectance contours are significantly similar in the same SMCF.

Figure 4c shows photographs of the SMCF in different diameters and underlying layers (The pristine photographs are shown in Fig. S12). The colors of the samples become more tunable for the nanowires of diameter less than 130 nm, whereas they do not show significant change for the nanowires of diameter greater than 130 nm. The reason for this lack of color change over 130 nm is that the scattering-based resonance, namely, the HE_11_ mode, occurs in the NIR region. To analyze the colors of the SMCFs quantitatively, the reflectance spectra are converted to the HSV color map (Fig. 4d,e)^35^. Figure 4d shows the Hue and Saturation of the measured reflectance spectra using color science. The symbol of ‘star’ indicates the tuning range of colors for the beneath layer (*i*.*e*., a-Si optical coating) with different thicknesses of a-Si of 10 nm, 15 nm, 20 nm, and 25 nm. The Hue is varied diversely as follows: 64° (*D*80) to 33° (*D*110) to 51° (*D*170) for SMCF (*t*10). 63° (*D*80) to 31° (*D*110) to 60° (*D*170) for SMCF (*t*15), 50° (*D*80) to 5° (*D*110) to 43° (*D*170) for SMCF (*t*20), 26° (*D*80) to −41° (*D*110) to 9° (*D*170) for SMCF (*t*25). The reversion of Hue is because of the HE_11_ resonance shift into the NIR region by increasing the diameter of Si NWA. Figure 4e represents the Values versus the diameter and thickness of the Si NWA and a-Si thin film, respectively. Interestingly, the valley of Value is found at the diameter of 110 nm except for the a-Si thickness of 25 nm. The Si NWA with the diameter of 110 nm eliminates the wavelength of ~580 nm, which is the most sensitive region of the human eye. Since the Value is proportional to the recognition of human eye, the dip of Value is observed at the diameter of 110 nm. Based on this reason, the Value of the a-Si thickness with 25 nm has relatively low, ~50%, because the a-Si layer suppresses the wavelength of 580 nm. At larger diameter of Si NWAs, the Value returns to the Values with the dimeter of 80 nm because the resonance is found in the NIR. All Values of SMCFs are considerably higher than the Value of Si NWA on PA as depicted in Fig. 2g.

**Figure 5:**
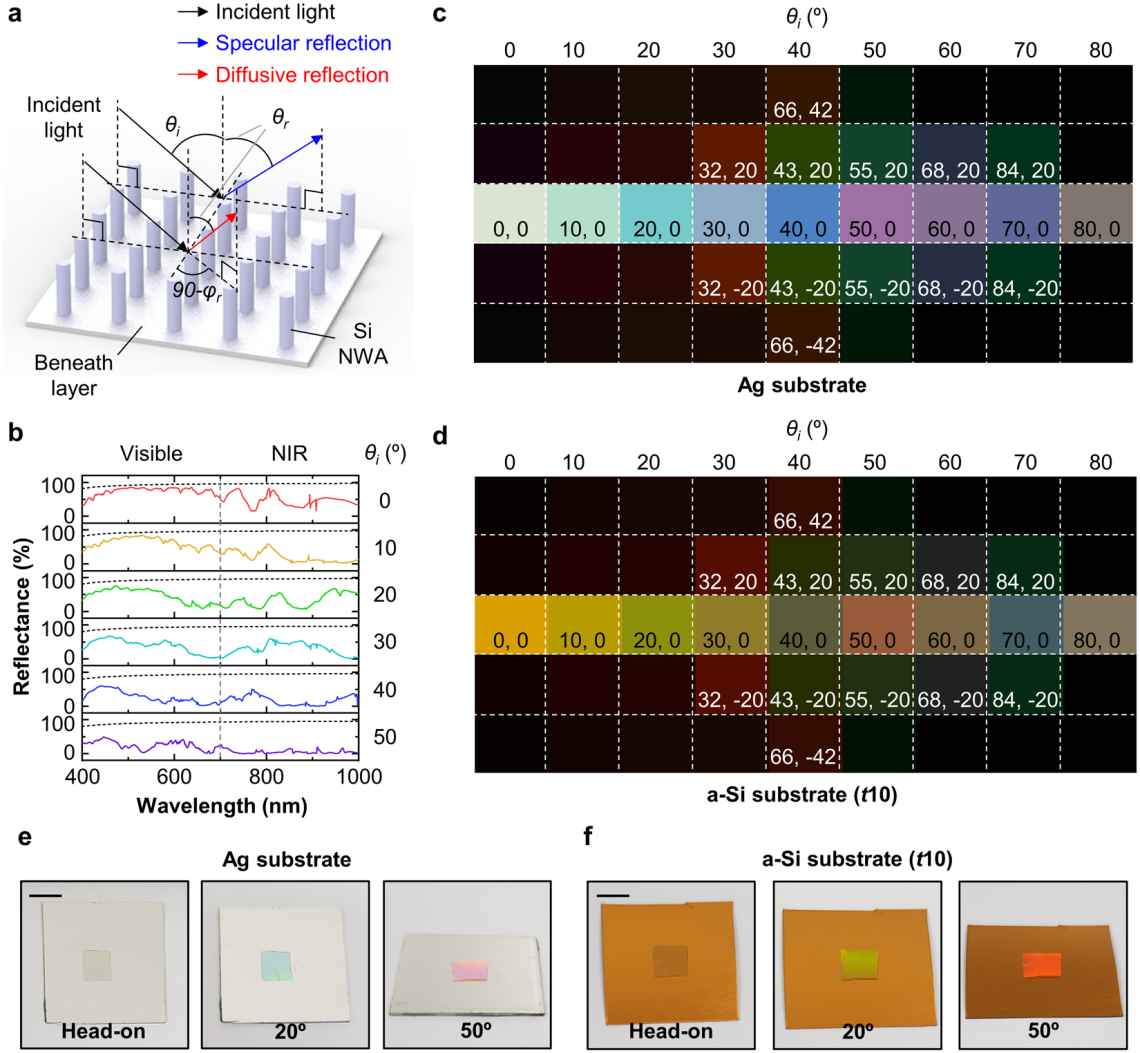
(**a**) Schematic illustration for angular response of Si NWA on beneath layer. The reflected light is classified with specular reflection and diffusive reflection. In the specular reflection, there is only one reflected angle, *θ*_*r*_, but, the diffusive reflection has two reflected angles such as *θ*_*r*_ and *φ*_*r*_. (**b**) Specular reflectance of Si NWA on Ag substrate as a function of wavelength and incident angle. The geometrical parameters of Si NWA are as follows: *D* = 150 nm, *Λ* = 1.25 μm, and *H* = 2 μm. The black dashed lines indicate the reflectance of Ag substrate. (**c**,**d**) Color representations of SMCF on Ag substrate and a-Si optical coating (*t*10). The texts in the color palette indicate the reflected angles, *θ*_*r*_ and *φ*_*r*_. (**e**,**f**) Photographs of SMCF on Ag substrate and a-Si optical coating as a function of viewing angle. The scale bar is 5 mm.

Figure 5a illustrates the angular response of Si NWA on beneath layer such as Ag and/or a-Si optical coating. Since Si NWA functions as a scatter shown in Fig. 3, the angular response of Si NWA should be studied. Basically, the periodically arranged nanostructures known as diffraction gratings reflect the incident light in diverse directions. In this situation, the reflected light is distinguished by two types such as specular reflection and diffusive reflection. The former is ruled by Snell’s law, and the latter is described by grating equation. In diffusive reflection, the reflected light propagates with two reflected angles such as *φ*_*r*_ and *θ*_*r*_, whereas specular reflection has one reflected angle, *θ*_*r*_ (*i*.*e*., *φ*_*r*_ = 0). Figure 5b exhibits the specular reflectance spectra of Si NWA (*D*150) on Ag substrate versus the incident angles. The resonance of Si NWA at normal incidence is found in the NIR region, hence, in visible range, the reflectance of Si NWA sustains Ag reflectance (*i*.*e*., gray dashed lines). As increased incident angle, the resonance of Si NWA is shifted to short wavelength. In other words, at normal incidence, SMCF with a large diameter is transparent and it intervenes color generation at oblique incident angles.

Figure 5c,d exhibit the color palettes of SMCF on Ag and a-Si optical coating (*t*15) with different incident angles. The texts in color palettes indicate the two reflected angles, *θ*_*r*_ and *φ*_*r*_. The black palettes mean no reflection. The colors by (0,0) reflected light (*i*.*e*., normally reflected light) show the colors of beneath layers such as the Ag and a-Si coating. By increasing the incident angle, the colors by specular reflection are remarkably changed in both two SMCFs, whereas the colors by diffusive reflection are the almost same with two SMCFs. This result presents that the color variation by specular reflection is started from the color of beneath layer, however, the colors by diffusive reflection are barely related to the color of beneath layers. Figure 5e,f demonstrate the color variation of SMCFs with two different beneath layers such as Ag and a-Si thin film. At normal incidence, both two SMCFs show the substrate colors, but the colors are totally different at two oblique angles (*i*.*e*., *θ*_*i*_, *θ*_*r*_ = 20 and 50°, *φ*_*r*_ = 0°). These colors are considerably similar to the simulation results.

**Figure 6:**
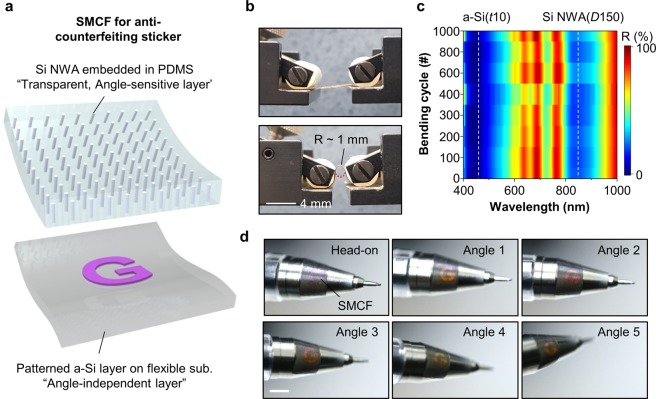
(**a**) Schematic illustration of anti-counterfeiting sticker by SMCF. (**b**) Photographs of SMCF in bending test. (**c**) Reflectance contour map of SMCF versus wavelength and bending cycle. The thickness and diameter of a-Si and Si NWA are 10 and 150 nm, respectively. The white lines indicate the resonant wavelengths of the SMCF. The bending radius is ~1 mm. (**d**) Photographs of SMCF as anti-counterfeiting sticker. Due to the angle sensitivity of Si NWA, the overall color of SMCF is varied according to the viewing angle. The scale bar is 5 mm.

Such iridescent color generation feature can be applied to an optical anti-counterfeiting system^36^. Particularly, the strong point of a-Si optical coating is angle-independent color filter (Fig. S13) and is easily capable of patterning by semiconductor processing. Thus, an anti-counterfeiting sticker can be implemented by using visibly transparent/angle-sensitive Si NWA embedded in PDMS and patterned/angle-independent a-Si layer on flexible substrate (Fig. 6a). To evaluate the mechanical stability, the bending test is conducted (Fig. 6b). The bending radius is ~1 mm, and bending cycle is 1000 times. The reflectance measurement is performed after 100 or 200 times folding (Fig. 6c). After 1000-folding, the resonant wavelengths are observed at ~450 and ~850 nm, which are the same as the first measurement. The SMCF used in measurement consists of Si NWA (*D*150) embedded in PDMS and a-Si (*t*10)/Ag (70 nm)/Polyester film (3 μm). Such outstanding mechanical property is because Si, which is inherently a hard material, is formed into an extremely thin wire and film. Figure 6d demonstrates the applicability of SMCF as the anti-counterfeiting sticker. The ‘G’ patterned anti-counterfeiting sticker is laminated on a pencil. The ‘G’ pattern engraved on the sticker at the front is purple corresponding to the color of 20 nm-thick a-Si layer on Ag (Fig. S2). As the pencil is tilted, the iridescent color variation is observed. In this case, the diffusive and specular reflected lights are collected together because the SMCF is attached on a curved surface. In addition to these mechanical and optical advantages, it is also expected that SMCF, based on structural color, is superior in durability in terms of UV degradation than general dye system.

In summary, we proposed and demonstrated the concept of a fine-tunable color filter, *i*.*e*., SMCF, with the Si NWA laminated on the a-Si/Ag film that provides color controllability based on the role of the scattered resonance mode (HE_11_ mode) from the Si NWA and the absorbing resonance mode (destructive interference) in the a-Si/Ag film. The measurement results for the Si NWA stacked on Ag and perfect absorber (PA) reveal that the HE_11_ mode of the Si NWA can be tuned not only by the variation of the diameters from 80 to 170 nm but also by the brightness of identical substrates. These reflectance spectra exhibit contradictory resonance features, such as a dip and a peak, depending on the substrates at the wavelength corresponding to HE_11_ modes. In addition to the reflectance tuning via the simple brightness difference of the substrate, the engineered underneath layer (*i*.*e*., a-Si on Ag) facilitates the fine tuning of the entire reflection spectrum through the base reflectance refinement of the underlying layer. Thorough theoretical analyses revealed that the two resonance of the SMCF are absorption-/scattering-based modes, respectively. With these two different resonance, the extended color tunability was achieved by adjusting the diameter of the SMCF. Both dips corresponding to the HE_11_ mode in Si NWA and the interference in the a-Si thin films were observed in the measured and simulated reflectance spectra from SMCF; hence, the linear combination of spectra extends the gamut in the color coordinate. Also, such the controllability of spectra contributes to the color generation depending on the viewing angles. Although a-Si layer and Si NWA have the conflicting features such as angle-independency and sensitivity, the colors for the viewing angles can be adjusted. Besides optical tunability, the structural softness of SMCF provides the applicability of flexible optical device such as optical anti-counterfeiting sticker. The SMCF on flexible film demonstrated an outstanding mechanical property and iridescent color generation, allowing the anti-counterfeiting sticker. We strongly believe that the enhanced color tunability and exceptional mechanical feature of the proposed structures find potential applications in pigment-free color printing, optical filters, flexible decorative devices and anti-counterfeiting systems.

## Methods

### Fabrication method for the vertical Si nanowire arrays

First, single crystalline Si wafers were thermally oxidized at 900 °C in H_2_ and O_2_ atmosphere at the ratio 14:8 for 90 minutes to form the SiO_2_ layer on the surface. To pattern the SiO_2_ nanodisk with the spacing of 1.25 μm, a positive photoresist was spin-coated onto the SiO_2_ layer, and then the lithography step was performed by using KrF scanner (PAS7000, ASML, Netherlands). The SiO_2_ nanodisks of different sizes from 200 to 290 nm were fabricated. Using this SiO_2_ nanodisk as a hard mask, the Si was anisotropically etched by using reactive ion etching (RIE), in which the mixtures of He, SF_6_, and O_2_ were used, to form the Si NWAs on the Si wafer. For the guided modes corresponding to the visible region, the diameters of Si nanowires were reduced to be in the range from 80 to 170 nm via an additional thermal oxidation process under the same conditions as above. After this process, the SiO_2_ shells of the nanowires were wet etched in an HF bath; the top SiO_2_ nanodisk was removed in this step.

### Transfer method of Si NWA to color films

To transfer the Si NWAs, the Si NWAs were embedded into polydimethylsiloxane (PDMS) with 5:1 ratio of base and agent. The mixture was poured and then spin-coated onto the wafer with the vertical Si NWAs at 1000 rpm for 60 s. The spin-coated PDMS film was cured at 230 °C on a hotplate for an hour. The cured PDMS film containing the Si NWA was detached by scraping the film from the substrate with a razor blade (BSC-21P, NT cutter, Japan). The detached PDMS embedded with the Si NWA was transferred to other color films. To detach Si NWA from Si substrate successfully, the direction and force that reacts to the razor blade are well aligned (Fig. S6a–c). Figure S6d,e show the SEM images of Si substrate after detaching Si NWA and bottom side of Si NWA embedded in PDMS. Because the remained parts of Si NWA are ~300 nm, the height of Si NWA embedded in PDMS is ~1.7 μm. As shown in the simulation result related to height effects of Si NWA, 1.7 μm-height Si NWA functions well as an optical filter.

### Fabrication method for the ultrathin absorbent color films

The ultrathin coloration film was deposited on a polycarbonate (PC) substrate by using an electron beam evaporator (KVE-E2000, Korea Vacuum Tech Ltd, Korea). A 5-nm-thick adhesion metal of Ti was first deposited, followed by 80 nm of Ag. Amorphous-Si (a-Si) with different thicknesses (*i*.*e*., 10, 15, 20, and 25 nm) was also deposited using the e-beam evaporator. Thickness monitoring was performed by a quartz crystal sensor during all the deposition processes. The Ti, Ag, and a-Si films were deposited at rates of ~0.5, ~1.0, and ~2.0 Å/s, respectively, at a pressure of ~3 × 10^−6^ Torr.

### Optical simulations

The rigorous coupled wave analysis (RCWA) method was used to calculate the specular and diffusive reflectance of the proposed photonic structures using commercial software (Diffract MOD, RSoft Design Group, USA). In the RCWA calculation, twenty diffraction orders and a grid size of 5 nm were used to calculate the diffraction efficiency; such simulation conditions are adequate to stabilize the results numerically. To find the scattered light, we used another mode based on 3-dimensional finite-difference time-domain (FDTD) in the commercial software (FullWAVE, RSoft Design Group, USA). In this simulation, the enclosed launch excitation type, which is one of the light source type in our simulation tool, was used. In the enclosed launch, only electric fields scattered by the structure propagate to outside. The unscattered light is absorbed at the boundary. Both scattered and unscattered fields are present within the enclosed launch boundary (Fig. S7). The boundary condition was defined as a periodic boundary condition in x-/y- directions and perfectly matched layer (PML) in z-direction. The mesh size was set to be 10 nm in the domain. The optical constants of a-Si, Ag, and Si were used from the library of the software. The refractive index of 1.43 was used for PDMS.

### Characterization

All reflectance values of the samples were characterized in the visible-near-infrared (NIR) wavelength range using an ultraviolet-visible-NIR spectrophotometer (Cary 5000 UV-Vis-NIR, Agilent Technologies, USA) with an unpolarized light source. A scanning electron microscope (SEM; S-4700, Hitachi High Technology, Japan) was used to observe the Si NWAs. Bending test on the SMCF was conducted with a motorized actuator (CONEX-LTA-HS, Newport) on an optical table. To evaluate the optical features of SMCF with stress, we established the measurement setup by using an optical fiber, spectrometer (QE65000, Ocean Optics, USA), a xenon-arc lamp (SLS401, Thorlabs, USA), and a motorized stage for bending (Fig. S7a). The white light is incident on the sample, and the reflected light is recollected by the optical fiber and reaches the spectrometer. Then, we can achieve the reflected light from SMCF. Last, the reflectance of the sample is obtained by processing with Ag reflectance as a reference.

## Supplementary information

Supporting Information
