# Supplementary material for: Reflective color filter with precise control of the color coordinate achieved by stacking silicon nanowire arrays onto ultrathin optical coatings
Source: Sci Rep. 2019 Mar 4;9:3350. doi: 10.1038/s41598-019-40001-1 (PMC6399234; doi:10.1038/s41598-019-40001-1)
Supplement: Supplementary file 1 — Supporting Information [file 41598_2019_40001_MOESM1_ESM.docx]

Supporting Information

**Reflective color filter with precise control of the color coordinate achieved by stacking silicon nanowire arrays onto ultrathin optical coatings**

*Han Sung Song^†^, Gil Ju Lee^†^, Dong Eun Yoo, Yeong Jae Kim, Young Jin Yoo, Dong-Wook Lee, Vantari Siva, Il-Suk Kang*, and Young Min Song**


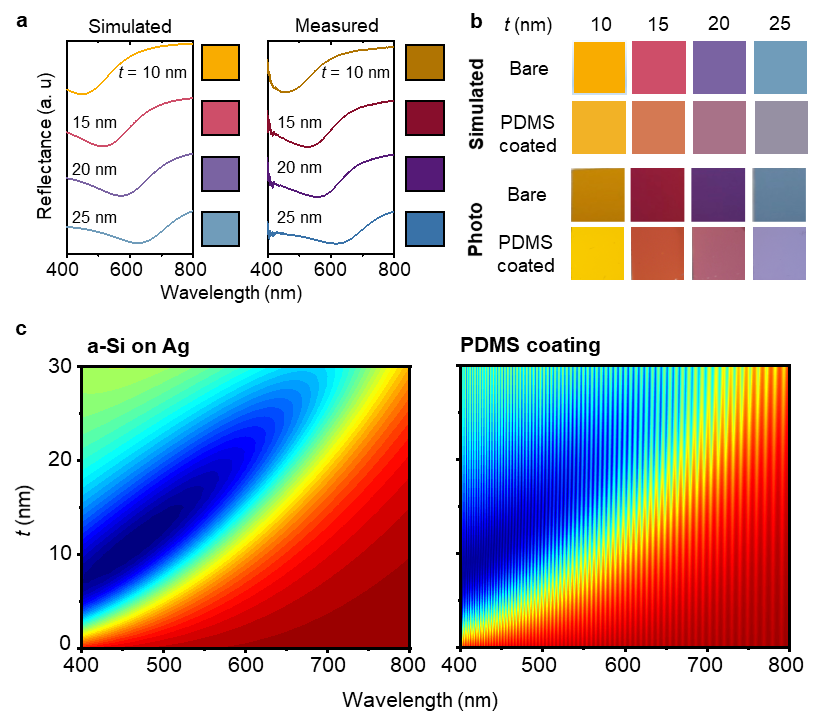


**Figure S1.** (a) Simulated and measured reflectance spectra of a-Si on Ag layer. *t* is the thickness of a-Si. The color representations are reproduced from the reflectances. (b) Comparison between the color representation from reflectance and photographs of the bare a-Si on Ag layers and the PDMS coated ones with the thicknesses of a-Si of 10, 15, 20, and 25 nm. (c) The contour plot of the simulated reflectance of the a-Si on Ag layers and the PDMS coated ones.


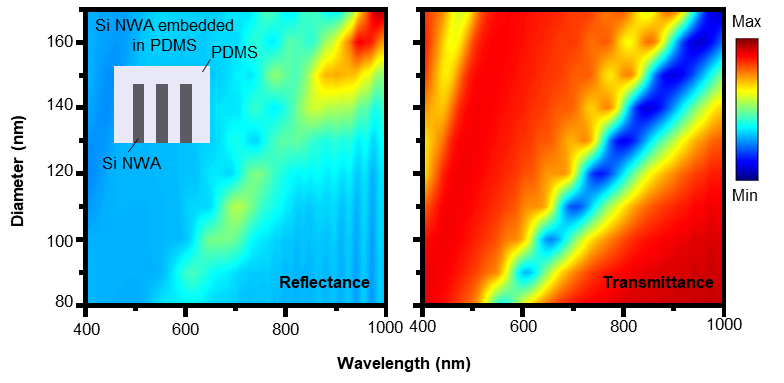


**Figure S2.** Contour plots of the total reflectance and transmittance of PDMS embedded Si NWA.


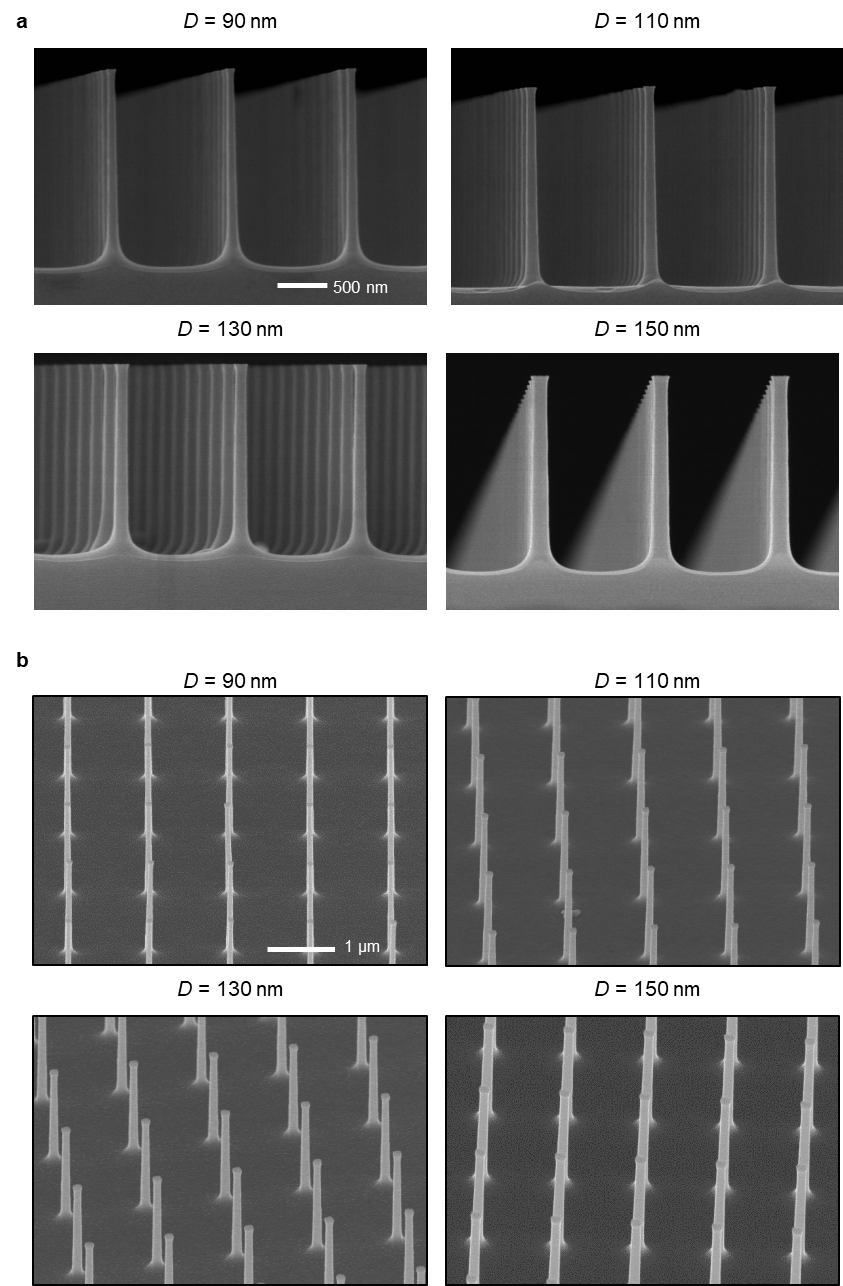


**Figure S3.** Scanning electron microscopy (SEM) images depending on the diameter: (a) cross-sectional view, (b) tilted-top view.

**
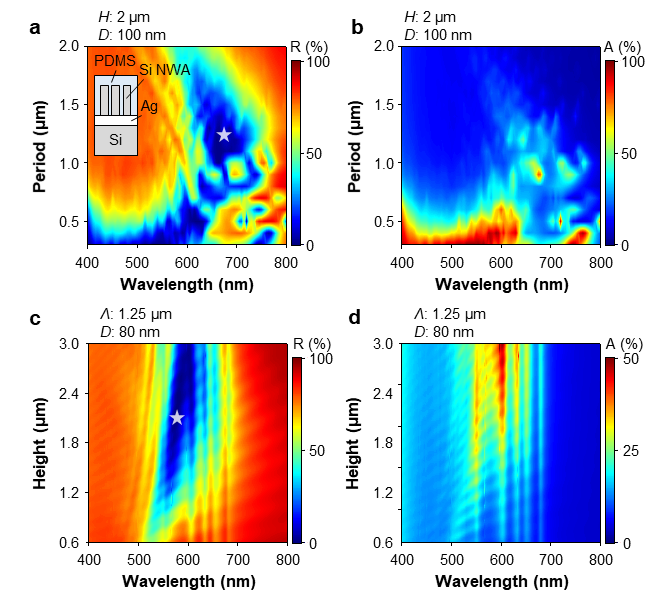
**

**Figure S4.** The influences of period and height in Si NWAs. (a,b) Contour plots of (a) specular reflectance and (b) absorptance for Si NWA as a function of period and wavelength with the diameter and height of 100 nm and 2 μm, respectively. (c,d) Contour plots of (c) specular reflectance and (d) absorptance for Si NWA versus height and wavelength with the diameter and period of 80 nm and 1.25 μm, respectively. The inset in **Figure S4**a displays the simulation structure. ‘Star’ symbols indicate the optimized points of period and height.

**
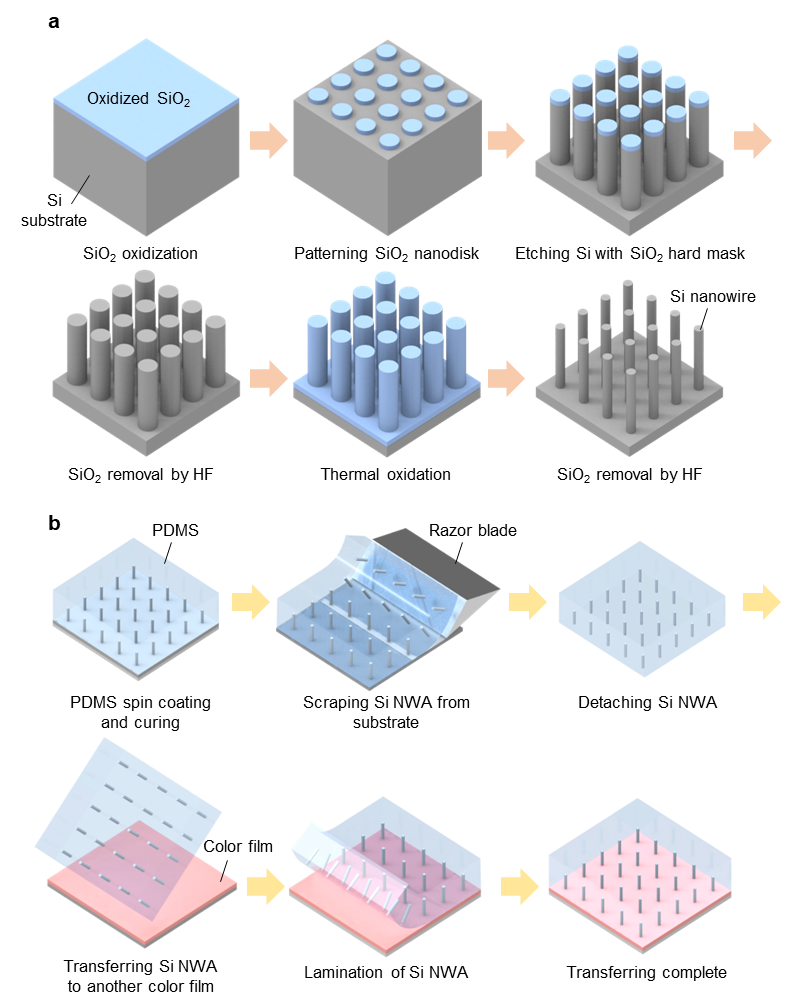
**

**Figure S5.** (a) Fabrication process of Si NWA via semiconductor processing. (b) The manufacturing process of the Si NWA embedded in PDMS.


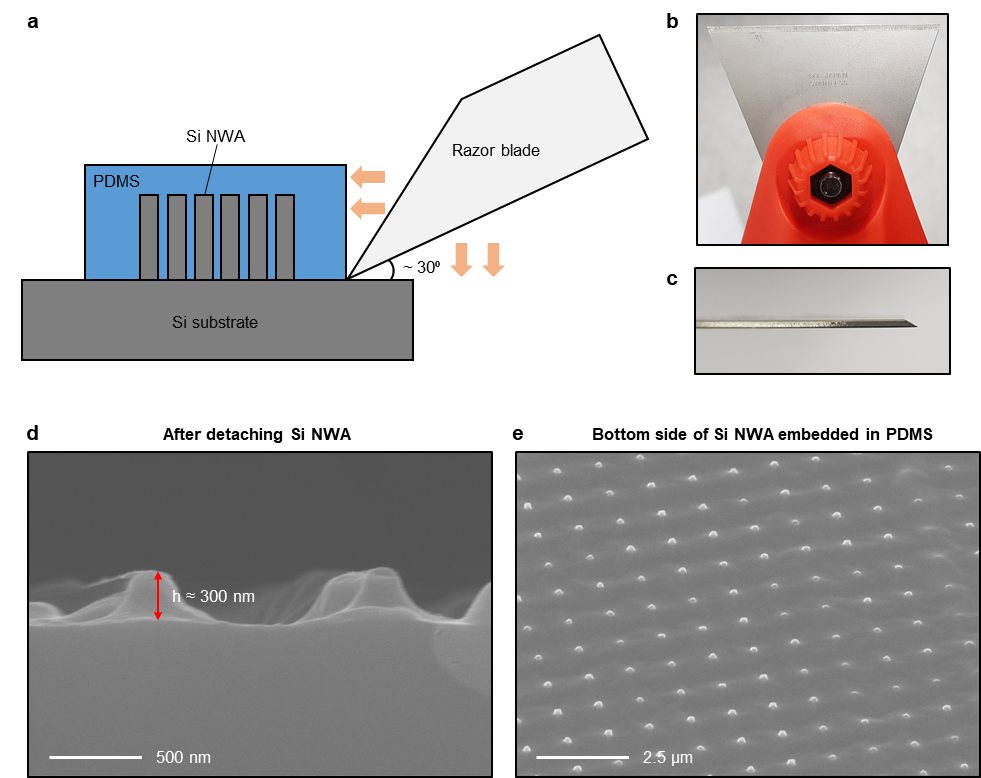


**Figure S6.** (a) Detailed schematic illustration of detaching process for Si NWA embedded in PDMS. The angle between Si substrate and razor blade is ~ 30°, and the force direction are indicated as orange arrows. (b,c) Photographs of razor blade used in our study. (d,e) SEM images of (d) Si substrate after detaching Si NWA and (e) bottom side of Si NWA embedded in PDMS


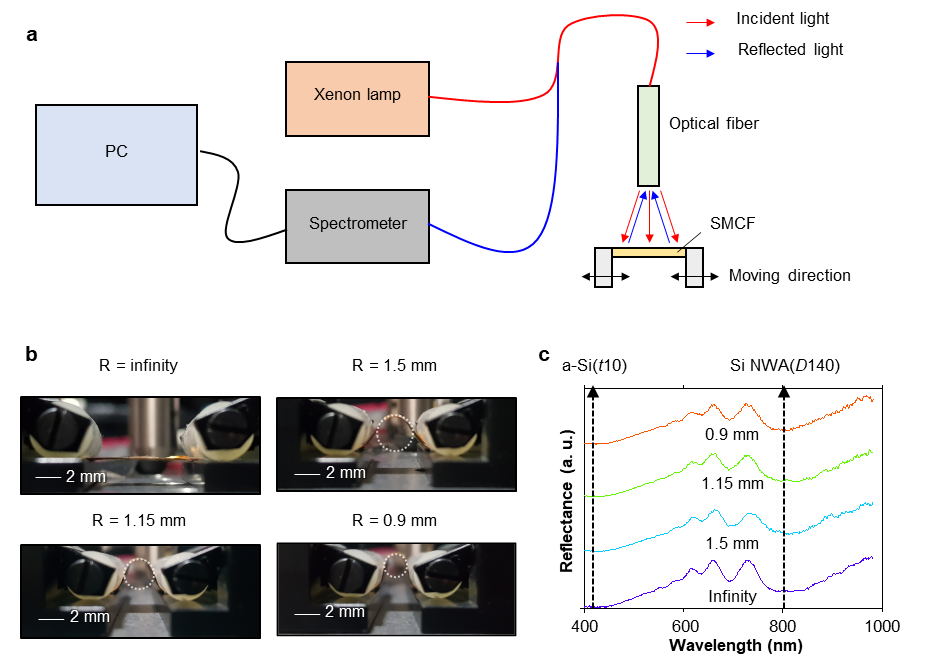


**Figure S7.** (a) Schematic illustration of measurement setup for reflectivity under bending status. (b) Photographs of bent SMCF. (c) Normalized reflectance spectra as a function of wavelength with four bending radii such as infinity, 1.5, 1.15, and 0.9 mm. The SMCF is composed of 10 nm-thick a-Si and Si NWA with the diameter, period, and height of 140 nm, 1.25 μm, and 2 μm.


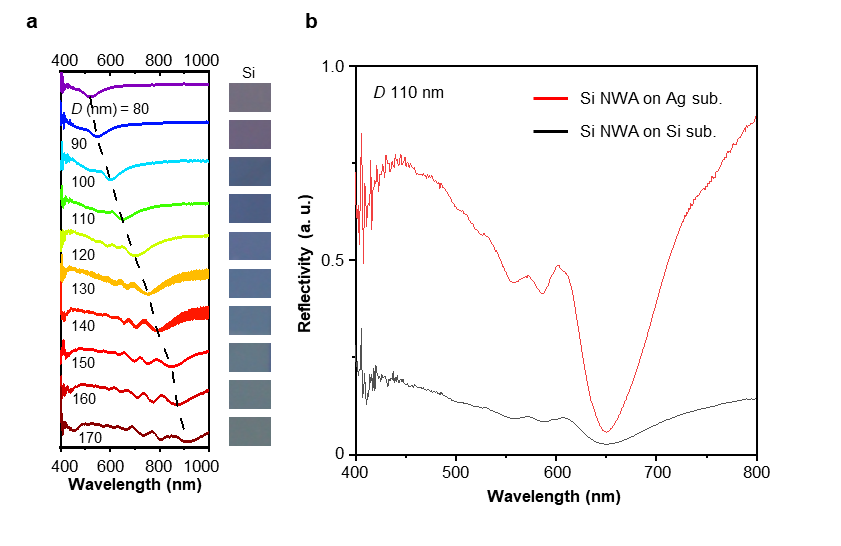


**Figure S8.** (a) Measured reflectance spectra of the Si NWA with a diameter ranging from 80 to 170 nm on Si substrate. The right color pallet is photographs of the Si NWAs on Si substrate corresponding to each diameter. (b) The absolute value of reflectance spectra for Si NWA on Si and Ag substrates.

**
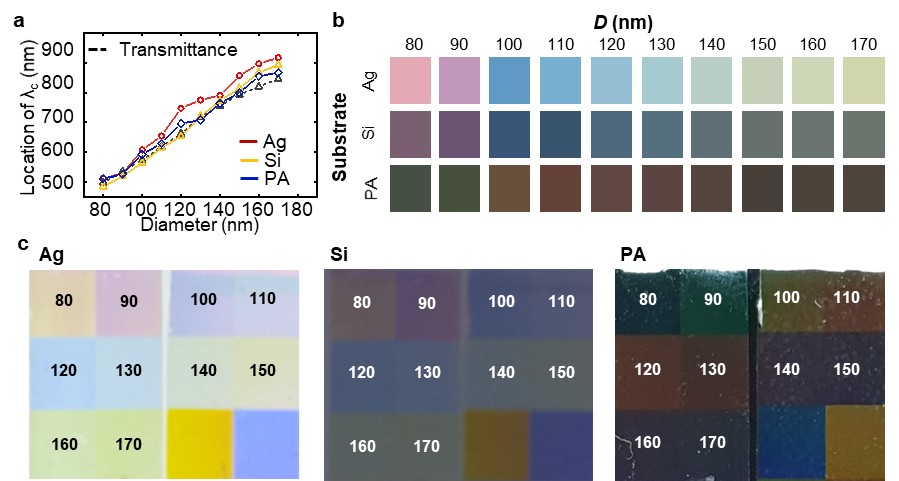
**

**Figure S9.** (a) Center wavelength of resonance points for the Si NWAs on Ag, Si, and PA substrates. All data are measured data. (b) Color representations from the reflectance of the Si NWAs on Ag, Si and PA substrates. (c) Photographs of the Si NWAs on Ag, Si and PA substrates.

**
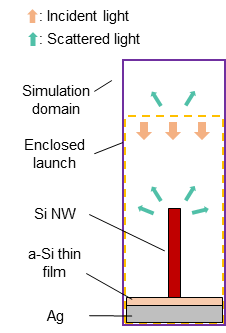
**

**Figure S10.** The schematic illustration of enclosed excitation type to observe only scattered light.


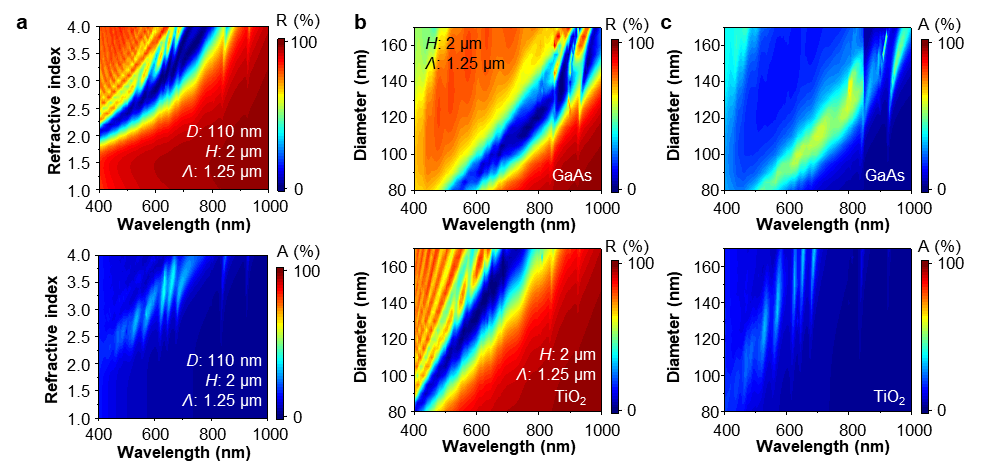


**Figure S11.** (a) Contour plots of nanowire array for (top) specular reflectance and (bottom) absorptance as a function of wavelength and refractive index. The geometrical parameters are as follows: *D* = 110 nm, *H* = 2 μm, and *Λ* = 1.25 μm. (b,c) Contour plots of (b) specular reflectance and (c) absorptance for nanowire arrays composed of (top) GaAs and (bottom) TiO_2_ as a function of wavelength and diameter. The other structural parameters are *H* = 2 μm, and *Λ* = 1.25 μm.


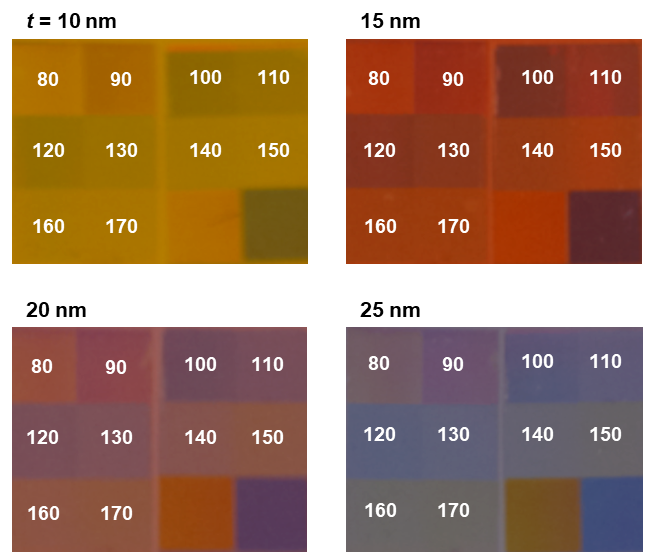


**Figure S12.** Photographs of SMCF. The white texts mean the diameter of Si NWA and the black texts are thicknesses of a-Si.


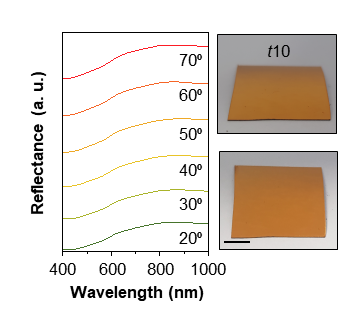


**Figure S13.** (left) Measured reflectance spectra of a-Si thin film (10 nm) on Ag as a function of wavelength and incident angle. (right) Photographs of a-Si thin film on Ag at (top) oblique angle and (bottom) normal angle. The scale bar is 5 mm.
